# Electron-Impact Cross Sections for Ground State to np Excitations of Sodium and Potassium

**DOI:** 10.6028/jres.109.037

**Published:** 2004-10-01

**Authors:** Philip M. Stone, Yong-Ki Kim

**Affiliations:** National Institute of Standards and Technology, Gaithersburg, MD 20899-8421

**Keywords:** core polarization, electron-impact, excitation cross section, potassium, sodium

## Abstract

Cross sections for electron impact excitation of atoms are important for modeling of low temperature plasmas and gases. While there are many experimental and theoretical results for excitation to the first excited states, little information is available for excitation to higher states. We present here calculations of excitations from the ground state to the np levels of sodium (*n* = 3 through 11) and potassium (*n* = 4 through 12). We also present a calculation for a transition from the excited sodium level 3*p* to 3*d* to show the generality of the method. Scaling formulas developed earlier by Kim [Phys. Rev. A **64**, 032713 (2001)] for plane-wave Born cross sections are used. These formulas have been shown to be remarkably accurate yet simple to use. We have used a core polarization potential in a Dirac-Fock wave function code to calculate target atom wave functions and a matching form of the dipole transition operator to calculate oscillator strengths and Born cross sections. The scaled Born results here for excitation to the first excited levels are in very good agreement with experimental and other theoretical data, and the results for excitation to the next few levels are in satisfactory agreement with the limited data available. The present results for excitation to the higher levels are believed to be the only data available.

## 1. Introduction

Our scaled Plane-Wave Born (PWB) cross sections for dipole- and spin-allowed excitation from the ground state of neutral hydrogen, helium, and lithium were presented in a recent paper in this journal [1]. In this paper, we use the same scaling methods to calculate excitation cross sections for neutral sodium and potassium from their ground states (3*s* ^2^S for sodium and 4*s* ^2^S for potassium) to the *np* ^2^P excited levels. The values of *n* are from *n* = 3 to 11 for sodium and *n* = 4 to 12 for potassium. Throughout the remainder of this paper we use the shorter notation of *ns* and *np* for these ^2^S and ^2^P levels.

The scaling method, developed by one of us [2], is applicable to allowed transitions (spin-allowed and dipole-allowed) of neutral atoms. The method uses two simple scaling formulas to convert PWB excitation cross sections into reliable cross sections comparable to the most accurate theoretical or experimental data available for dipole-allowed transitions. The PWB cross sections are calculated from uncorrelated wave functions, and the scaling requires only the binding energy *B* of the bound electron that is excited, the excitation energy *E*, and an accurate dipole oscillator strength *f* for the transition. Simplicity of the method to scale PWB cross sections allows the user to generate a large number of cross sections reliably and quickly. This is particularly useful for modeling of low temperature plasmas and hot gases, where a large number of excitation cross sections is usually required.

Polarization of the core electrons by the valence electron is important in alkali metal atoms. We treat the long-range effect of core polarization by including a polarization potential explicitly in our calculation of the wave functions of the target atom and we add a term to the dipole-moment operator when calculating the oscillator strength of the transition. The core polarization effect is not very noticeable for sodium but is large for potassium.

## 2. Outline of Theory

The scaling methods for spin-allowed and dipole-allowed transitions are described in earlier papers [1,2]. The first scaling method, the BE scaling, replaces the incident electron energy *T* in the denominator of the PWB cross section by *T* + *B* + *E*, i.e.,

$$\sigma_{\text{BE}}=\sigma_{\text{PWB}}\left[T/\left(T+B+E\right)\right].$$ (1)

This scaling is similar to a scaling for ionization cross sections used earlier by Burgess [3], who shifted the incident energy *T* by *B* + *U*, where *U* is the kinetic energy of the target electron. However, in the BE scaling adopted by Kim [2] for excitation cross sections, *T* is shifted by *B* + *E*. The BE scaling not only changes the magnitude but also the shape of the original PWB cross sections. The BE scaling corrects the deficiency in the collision theory; i.e., the use of the PWB approximation.

The second scaling formula, the *f* scaling, multiplies the entire cross section by the ratio of an accurate *f* value to the less accurate *f* value calculated by the actual wave functions used to generate the unscaled PWB cross sections:

$$\sigma_{f}=\left(f_{\text{accu}}/f_{\text{sc}}\right)\sigma_{\text{PWB}}.$$ (2)

where *f*_sc_ is the single configuration (or uncorrelated) *f* value and *f*_accu_ is the more accurate value obtained from correlated (or multiconfiguration) wave functions or from a reliable experiment. Accurate *f* values are frequently available [4].

The *f* scaling compensates for the inadequacy of the wave functions when electron correlation is significant. In principle, the *f* scaling should compensate for the rather large core polarization effect in the alkali metal atoms. Because the *f* scaling is such a large correction for potassium, however, we chose to include core polarization explicitly in calculating the PWB cross sections that are our starting values. We use only single configuration wave functions and apply *f* scaling. With core polarization, the calculated *f* values are closer to accurate experimental values but the *f* scaling still gives a noticeable correction.

The BE and *f* scaling may be applied consecutively, i.e.,

$$\sigma_{\text{BE}f}=\left(f_{\text{accu}}/f_{\text{sc}}\right)\sigma_{\text{BE}},$$ (3)

where *σ*_BE_ is the BE-scaled PWB cross section calculated from single-configuration wave functions.

Kim has shown many examples [2] in which the BE scaling alone or in combination with the *f* scaling transformed PWB cross sections for dipole-allowed and spin-allowed excitations into reliable cross sections comparable to the convergent close coupling (CCC) method [5] or accurate experiments.

Resonances in the electron-impact excitation cross sections of atoms in the vicinity of the excitation thresholds cannot be accounted for by first-order perturbation theories such as the PWB approximation. Hence the present scaled cross sections do not exhibit any resonances.

Polarization of the core electrons by valence electrons affects the potential in which the valence electrons move. This is especially important in alkali metals where the one valence electron is particularly sensitive to any core changes. The polarization can be represented by a classical model and was first used in quantum mechanical calculations by Biermann [6], although the origin of the core-polarization picture can be traced back even to the semiclassical study of the helium atom by Heisenberg [7] in 1926.

The most complete quantum approach to the core-polarization effect was presented by Böttcher and Dalgarno [8]. Their theory, which is based on a perturbation approach, leads to asymptotic forms of the core polarization potential *V*_p_ and corrected dipole transition moment ***d***_eff_ which are in agreement with the classical formulas.

Neither classical nor quantum mechanical approaches provide the form of the core-polarization corrections for the small or intermediate *r* region. Moreover, the asymptotic form for large *r* diverges for *r* → 0. Therefore, there is a need to introduce an arbitrary cutoff function to remove this divergence at *r* = 0.

Migdalek and Baylis [9] proposed to introduce a cutoff function directly into the expression for the effective field ***E***_v_ produced by a valence electron at the core, and subsequently obtained the following expressions for *V*_p_ and ***d***_eff_:

$$V_{p}=-\frac{\alpha_{d}r^{2}}{2{\left(r^{2}+r_{c}^{2}\right)}^{3}},$$ (4)

$$d_{\text{eff}}=-r\left[1-\frac{\alpha_{d}}{{\left(r^{2}+r_{c}^{2}\right)}^{3/2}}\right],$$ (5)

where *α*_d_ is the static dipole polarizability of the core and *r*_c_ is the cutoff radius. Equation (5) can also be used to correct the ***r*** in the operator exp(*i****K*** · ***r***) of the matrix element for the generalized oscillator strength. In other words, the correction for the dipole transition operator can also be used to introduce the correction for the core polarization in plane-wave Born cross sections.

The core-polarization potential *V*_p_, Eq. (4), as well as the matching form of the dipole transition operator, Eq. (5), were introduced into a Dirac-Fock wave function code to account for the core-polarization effect in wave functions and the dipole transition operator. The polarizabilities used were *α*_d_ = 0.9457 $a_{0}^{3}$ for Na and 5.457 $a_{0}^{3}$ for K, where *a*_0_ is the Bohr radius (0.529 Å). The cutoff radii were *r*_c_ = 0.7967 *a*_0_ and 1.433 *a*_0_, respectively.

## 3. Results

**Fig. 1:**
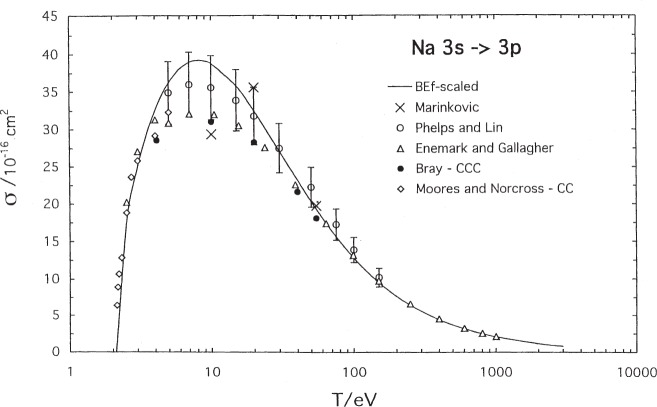
Sodium 3*s*–3*p* electron-impact excitation cross sections. The solid curve is our scaled plane-wave Born (PWB) result, the solid circles are accurate theoretical results from the convergent close coupling (CCC) method of Bray [12], the diamonds are theoretical results from the close coupling calculation of Moores and Norcross [15], the open circles are experimental results of Phelps and Lin [13] that include corrections for cascading from the upper levels, and the triangles are experimental results of Enemark and Gallagher [14] that are also corrected for cascading. The crosses are experimental results of Marinkovic et al. [29].

**Fig. 2:**
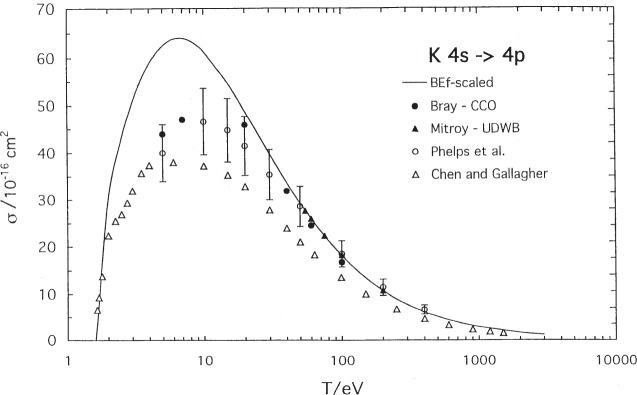
Potassium 4*s*–4*p* electron-impact excitation cross sections. The solid curve is our scaled plane-wave Born (PWB) result, the solid circles are theoretical results from the coupled-channel optical (CCO) method of Bray et al. [16], the open circles are experimental results of Phelps et al. [17] that include corrections for cascading from the upper levels, and the grey triangles are experimental results of Chen and Gallagher [19] that do not correct for cascading. The solid triangles are theoretical calculations of Mitroy [18] using a unitarized form of the distorted wave Born approximation.

**Fig. 3:**
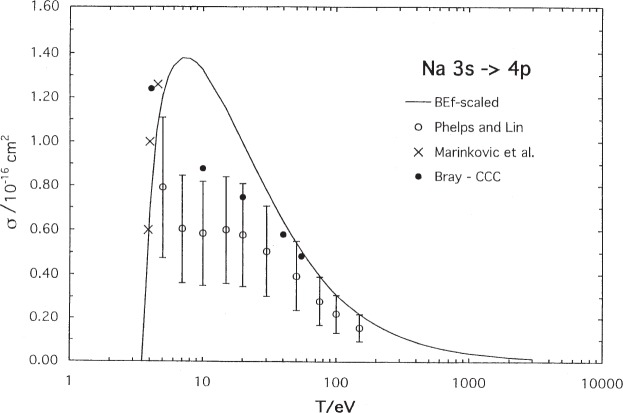
Sodium 3*s*–4*p* electron-impact excitation cross sections. The measurements of Marinkovic, Wang, and Gallagher [20] are shown. The other symbols are as in Fig. 1.

**Fig. 4:**
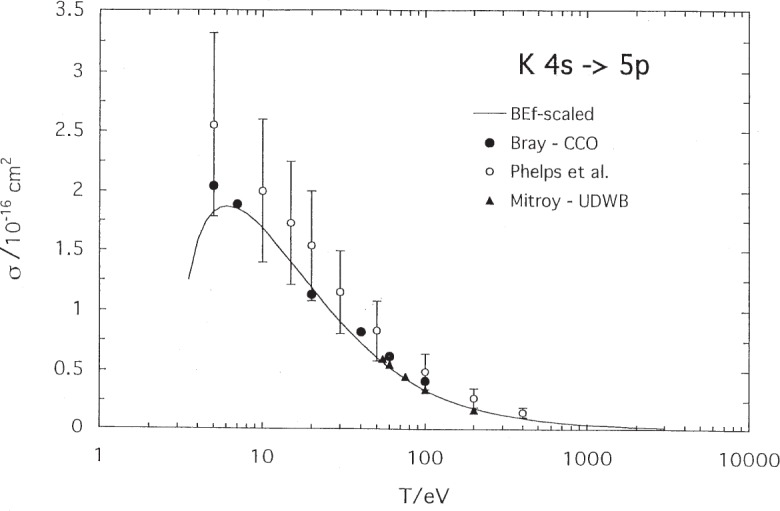
Potassium 4*s*–5*p* electron-impact excitation cross sections. The symbols are as in Fig. 2.

**Fig. 5:**
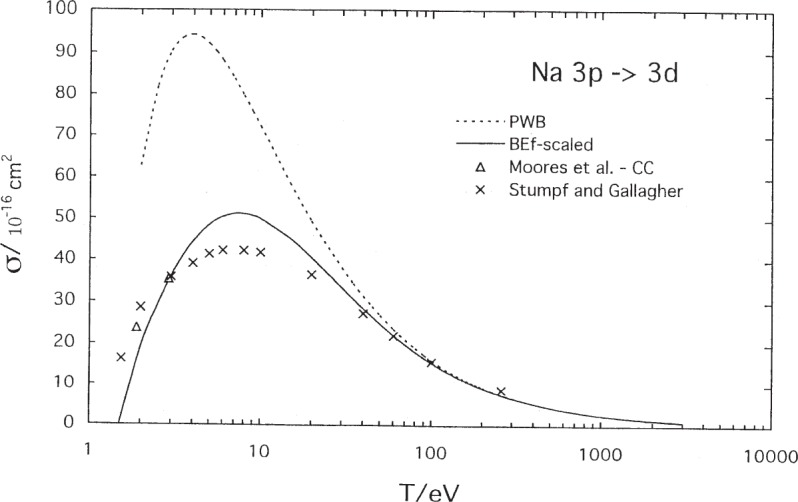
Sodium 3*p*–3*d* electron-impact excitation cross sections. The solid curve is our scaled plane-wave Born (PWB) result for excitation, while the dotted curve is the PWB (unscaled) cross section. The crosses are experimental results of Stumpf and Gallagher [21] and diamonds are the calculated results of Moores et al. [22].

**Fig. 6:**
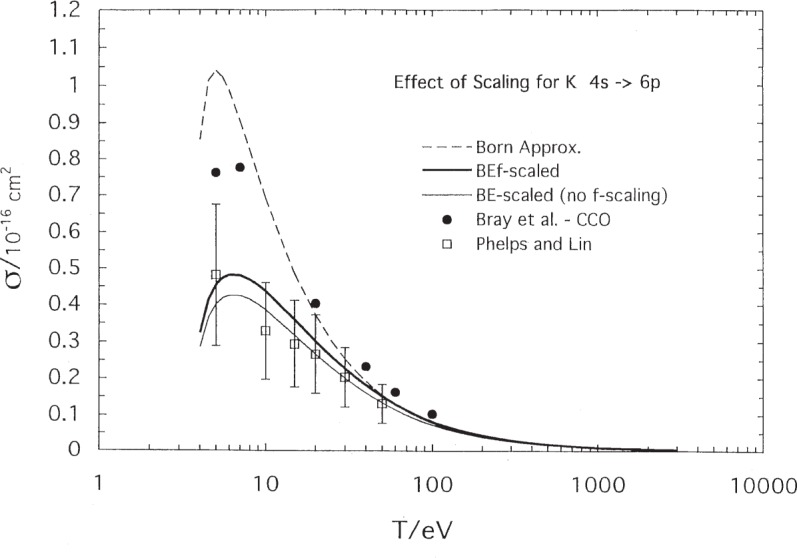
This figure shows the magnitude of the scaling effect on plane-wave Born cross sections. BE-scaling, Eq. (1), for the 4*s*–6*p* transaction of potassium decreases the Born cross section by a factor of nearly 3 in the peak region. Adding the *f*-scaling, Eq. (2), raises the cross section back up by about 10 %. Experimental measurements of Phelps and Lin [13] and calculated couple-channel optical results of Bray et al. [16] are also shown.

**Fig. 7:**
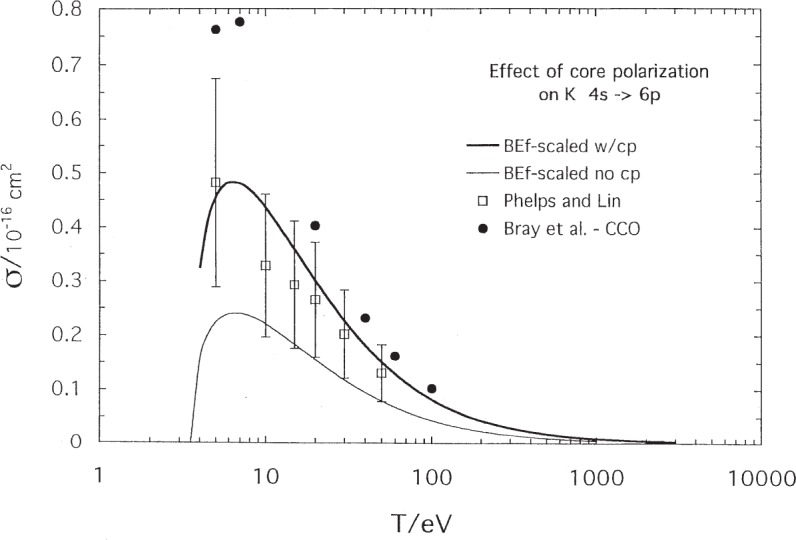
The effect of core polarization on the potassium 4*s*–6*p* transition is to raise the cross section by about a factor of 2 in the peak region.

We present the calculated cross sections for sodium and potassium in Tables 1 and 2. Our PWB cross sections were generated from single configuration Dirac-Fock wave functions. The calculated cross sections are compared to other theories and experiments in Figs. 1–7.

The numerical data in Tables 1 and 2 can be extended to higher incident energies by using the well known Bethe formula [10] for the plane-wave Born approximation for fast (but nonrelativistic) incident electrons. In our notation, the asymptotic expression becomes:

$$\sigma_{\text{asympt}}\left(T\right)=\frac{4\pi a_{0}^{2}R}{T+B+E}\left[a\ln\left(T/R\right)+b+cR/T\right]\left(f_{\text{accu}}/f_{\text{sc}}\right),$$ (6)

where *a*, *b*, and *c* in the square brackets are dimensionless constants, *T* is the incident electron energy, and *R* is the Rydberg energy (13.61 eV). Equation (6) should be used for *T* > 3 keV. The values of *a*, *b*, and *c* are included in Tables 1 and 2. Note that a relativistic form of the Bethe formula [11] should be used for *T* > 10 keV. Also, *B* + *E* can be omitted in the denominator at this high range of *T*.

The results for excitation of the first resonance line of sodium is shown in Fig. 1. BE*f*-scaled results agree well with the accurate CCC calculations of Bray [12] and the careful experimental values of Phelps and Lin [13]. Phelps and Lin corrected their optical excitation data for cascading from higher levels using calculated transition probabilities that are in good agreement with experimental values. At low energies, just above the threshold for excitation, our results are in very good agreement with the optical excitation measurements of Enemark and Gallagher [14] and with the close coupling calculations of Moores and Norcross [15].

The situation is nearly as good for excitation of the first resonance line of potassium, shown in Fig. 2. In this case, we agree with the close coupling optical (CCO) calculations of Bray et al. [16] and the experimental measurements of Phelps et al. [17] above about 30 eV, but we are high in the region of the peak cross section by about 30 %. The unitarized distorted wave Born calculations of Mitroy [18] above 54 eV are also shown, and agree with the other values. We are in very good agreement with the measurements of Chen and Gallagher [19] in the threshold region.

The lowest excitations of Na and K have very large cross sections compared to higher excitations, reflecting the fact that the *f* values for the former excitations are almost unity, leaving very little for the higher excitations.

Excitation to the next resonance levels of sodium and potassium are shown in Figs. 3 and 4. In these cases, our BE*f*-scaled results differ significantly in the peak region from the optical excitation results [13,17] and the CCC [12] and the CCO [16] calculations respectively. Our Na calculation, however, agrees well with the threshold measurements of Marinkovic, Wang, and Gallagher [20]. Results for excitation to higher resonance levels are given in Tables 1 and 2.

While not the purpose of this paper but as an additional verification of the validity of the calculational technique, we have compared the BE*f*-scaled calculation with experimental data for excitation from the 3*p* excited level of Na to the 3*d* level. The results are shown in Fig 5. This figure also shows the unscaled plane-wave Born (PWB) result so that the magnitude of our scaling is evident. Agreement is good with the measurements of Stumpf and Gallagher [21] and the close coupling results of Moores et al. [22]. Stumpf and Gallagher actually measured from laser excited 3 ^2^P_1/2_ levels. The measurements were corrected for cascading and polarization of the emitted fluorescence using Born approximation results and normalized at high energies to the Born approximation value. The Born approximation is not valid at energies below about 300 eV but was used nevertheless for the corrections. They used the Born-Ochkur approximation for the polarization factor. The 3 ^2^P_1/2_ measurements were converted to 3*p* to 3*d* excitation by use of Born approximation results.

Figure 6 shows the effect of our scaling formulas on the Born cross section. The figure is for potassium 4*s*–6*p* excitation where core polarization is a large effect. The BE-scaling, Eq. (1), makes the largest correction to the Born cross section, decreasing it by a factor of more than 2 in the peak region. Adding the *f*-scaling, Eq. (2), then raises the cross section by about 10 %.

Figure 7 shows the effect of the core polarization on the potassium 4*s*–6*p* excitation. The cross section with polarization is larger by about a factor of 2 in the peak region.

**Fig. 8:**
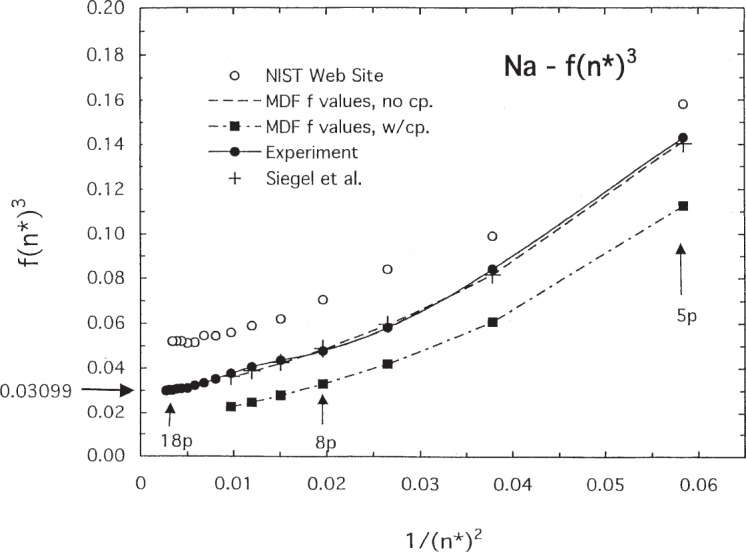
Values of *f ·* (n*)^3^ for the resonance transitions 3*s–np* for sodium, showing the values for experimental data (solid circles) as described in the text and for the calculated results of Siegel et al. [23] (plus signs). The extrapolated experimental value at the ionization limit (n* → ∞) is 0.0305, in agreement with photoionization measurements of Marr and Creek [31] (arrow on ordinate). Also shown are the results from the single configuration Dirac-Fock calculation of the present work (solid squares) and values from the current NIST web site [4] (open circles). The NIST web site values are from earlier measurements than the more recent experimental data shown in the plot.

**Fig. 9:**
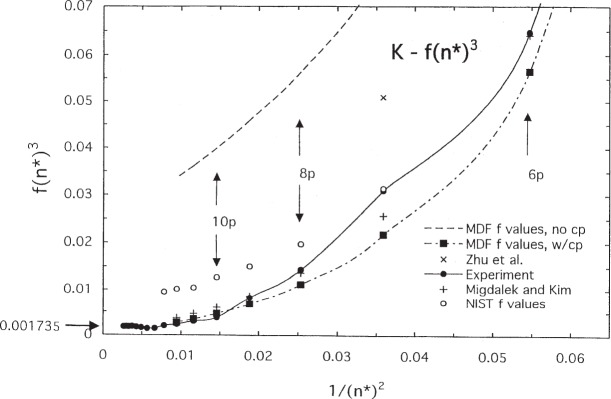
Values of *f ·* (n*)^3^ for the resonance transitions 4*s*–*np* for potassium, showing the values for experimental data as described in the text (solid circles) and for the calculated results of Migdalek and Kim [24] (plus signs). The extrapolated experimental value at the ionization limit (n* → ∞) is 0.00177, while the photoionization measurements of Marr and Creek [31] give 0.001735 (arrow on ordinate). Also shown are the results from the single configuration Dirac-Fock calculation of the present work (solid squares) and values from the current NIST web site [4] (open circles). The recent calculations of Zhu et al. [30] (crosses) are too high compared to the experimental data. The NIST web site values are from earlier measurements than the more recent experimental data shown in the plot. The calculated values of Migdalek and Kim, incorporating a polarization potential, are in good agreement with the experimental values.

The effect of core polarization (cp) on the *f* values is small for sodium but large for potassium. This is evident in Figs. 8 and 9 where we have plotted *f* · (n*)^3^ versus 1/(n*)^2^, where n* is the effective principal quantum number for the upper state. The change in *f* · (n*)^3^ for potassium (Fig. 9) is much larger than for sodium (Fig. 8). Quantum defect theory predicts that *f* · (n*)^3^ should extrapolate at high n* to a constant value at the ionization threshold and connect smoothly to a value determined from the photoionization cross section. Extrapolation of the experimental values of *f* · (n*)^3^ to the ionization threshold *(n* → ∞) gives *f* · (n*)^3^ = 0.0305 for Na and 0.00177 for K. These values agree well with independently measured values from photoionization cross sections of Marr and Creek [31] which give 0.03099 and 0.001735, respectively. The Marr and Creek values are shown in Figs. 8 and 9 as arrows on the left-hand axis. The *f* values on the NIST website [4] are not in good agreement with the latest experiments. The effect of core polarization on the *f* values is about 25 % for sodium but a factor of 5 for potassium.

We have used the accurate calculated *f* values of Siegel et al. [23] for the *f* scaling of Na, and values from the similar calculation of Migdalek and Kim [24] for the *f* scaling of K. These results differ by a negligible amount from experimental *f* values shown in Figs. 8 and 9. We have used these calculated values, rather than the experimental values, because they are available for the fine structure components, while the best experimental values generally do not distinguish the components. For Na, the experimental *f* values shown in Fig. 6 are the measurements of Filippov and Prokoviev [25] for *n* = 3 to *n* = 8, normalized to the 3*s*–3*p* measurement of Volz et al. [26]. For *n* = 9 to 24, the Na values are from recent measurements of Nawaz et al. [27]. For potassium, the experimental *f* values shown in Fig. 7 are from Shabanova and Khlyustalov [28] for *n* = 4 to 14, and Nawaz et al. [27] for higher *n*.

The cross sections for excitation to the higher *np* levels are converging to values that scale as (n*)^3^. A good estimate for cross sections to higher levels than presented here is to scale the cross sections for *n* = 11 of Tables 1 and *n* = 12 of Table 2 by the ratio of the n* values raised to the third power.

Accurate wave functions are particularly necessary when the *f* values are very small, as is the case when the Cooper minimum is near (below or above) the ionization threshold. The Cooper minimum occurs at an energy where the dipole matrix element goes to zero because positive and negative contributions arising from overlap of the lower and upper state wave functions cancel each other. The Cooper minimum is particularly evident for alkali atoms because of core polarization. The minimum appears closer to the ionization limit in K than in Na because of the latter's larger core polarization.

## Figures and Tables

**Table 1:** Excitation cross sections for dipole- and spin-allowed excitations from the ground state of Na. Excitation energies *E* in eV, dipole *f* values from uncorrelated wave functions (*f*_sc_), accurate *f* values (*f*_accu_) by Siegel et al. [23], and BE*f*-scaled excitation cross sections *σ*_BE_*_f_* in Å^2^ as functions of incident electron energy *T* in eV. The initial state is 3*s* ^2^S. The experimental ionization energy *B* = 5.139077 eV has been used in the scaling. The constants *a*, *b*, and *c* of Eq. (6) are included

Final state	3*p*	4*p*	5*p*	6*p*	7*p*	8*p*	9*p*	10*p*	11*p*
*E*	2.104E+00	3.753E+00	4.345E+00	4.624E+00	4.778E+00	4.872E+00	4.934E+00	4.976E+00	5.007E+00
*f*_sc_	9.650E−01	1.180E−02	1.591E−03	4.480E−04	1.810E−04	9.100E−05	5.200E−05	3.300E−05	2.200E−05
*f*_accu_	9.720E−01	1.331E−02	1.980E−03	6.020E−04	2.580E−04	1.340E−04	7.900E−05	5.100E−05	3.500E−05
Const. *a*	6.241E+00	4.276E−02	4.981E−03	1.319E−03	5.160E−04	2.530E−04	1.430E−04	8.900E−05	5.900E−05
Const. *b*	1.611E+01	5.477E−01	1.396E−01	5.822E−02	3.037E−02	1.802E−02	1.163E−02	7.972E−03	5.714E−03
Const. *c*	−2.062E−01	−4.093E−02	−8.539E−03	−3.035E−03	−1.417E−03	−7.780E−04	−4.750E−04	−3.130E−04	−2.180E−04
*T*	*σ*_BE_*_f_*	*σ*_BE_*_f_*	*σ*_BE_*_f_*	*σ*_BE_*_f_*	*σ*_BE_*_f_*	*σ*_BE_*_f_*	*σ*_BE_*_f_*	*σ*_BE_*_f_*	*σ*_BE_*_f_*

2.102298	0								
2.5	1.774E+01								
3.0	2.508E+01								
3.5	2.954E+01								
4.0	3.257E+01	6.921E−01							
4.5	3.471E+01	1.050E+00							
5.0	3.624E+01	1.209E+00	2.882E−01	1.072E−01	4.788E−02	2.325E−02			
5.5	3.733E+01	1.294E+00	3.370E−01	1.418E−01	7.414E−02	4.395E−02	2.832E−02	1.936E−02	1.384E−02
6.0	3.811E+01	1.342E+00	3.613E−01	1.573E−01	8.479E−02	5.154E−02	3.389E−02	2.355E−02	1.706E−02
7	3.898E+01	1.379E+00	3.802E−01	1.691E−01	9.261E−02	5.699E−02	3.781E−02	2.646E−02	1.927E−02
8	3.925E+01	1.376E+00	3.821E−01	1.709E−01	9.399E−02	5.801E−02	3.857E−02	2.703E−02	1.971E−02
9	3.916E+01	1.356E+00	3.768E−01	1.688E−01	9.297E−02	5.744E−02	3.822E−02	2.680E−02	1.955E−02
10	3.883E+01	1.326E+00	3.682E−01	1.650E−01	9.093E−02	5.619E−02	3.740E−02	2.623E−02	1.913E−02
15	3.581E+01	1.150E+00	3.162E−01	1.414E−01	7.781E−02	4.806E−02	3.198E−02	2.243E−02	1.636E−02
20	3.251E+01	9.967E−01	2.717E−01	1.211E−01	6.658E−02	4.109E−02	2.733E−02	1.916E−02	1.397E−02
25	2.960E+01	8.758E−01	2.371E−01	1.054E−01	5.790E−02	3.572E−02	2.375E−02	1.665E−02	1.214E−02
30	2.712E+01	7.801E−01	2.100E−01	9.321E−02	5.114E−02	3.154E−02	2.096E−02	1.469E−02	1.071E−02
35	2.503E+01	7.032E−01	1.884E−01	8.348E−02	4.577E−02	2.821E−02	1.875E−02	1.314E−02	9.577E−03
40	2.324E+01	6.400E−01	1.708E−01	7.557E−02	4.141E−02	2.552E−02	1.695E−02	1.188E−02	8.658E−03
45	2.170E+01	5.875E−01	1.562E−01	6.903E−02	3.780E−02	2.329E−02	1.547E−02	1.084E−02	7.899E−03
50	2.037E+01	5.430E−01	1.439E−01	6.352E−02	3.477E−02	2.142E−02	1.422E−02	9.963E−03	7.262E−03
60	1.816E+01	4.719E−01	1.243E−01	5.479E−02	2.997E−02	1.845E−02	1.225E−02	8.580E−03	6.253E−03
70	1.641E+01	4.176E−01	1.095E−01	4.818E−02	2.634E−02	1.621E−02	1.076E−02	7.534E−03	5.490E−03
80	1.500E+01	3.747E−01	9.787E−02	4.300E−02	2.349E−02	1.445E−02	9.591E−03	6.715E−03	4.893E−03
90	1.382E+01	3.400E−01	8.849E−02	3.884E−02	2.120E−02	1.304E−02	8.653E−03	6.058E−03	4.414E−03
100	1.283E+01	3.114E−01	8.077E−02	3.541E−02	1.932E−02	1.188E−02	7.883E−03	5.518E−03	4.020E−03
110	1.198E+01	2.873E−01	7.431E−02	3.254E−02	1.775E−02	1.091E−02	7.239E−03	5.067E−03	3.691E−03
120	1.125E+01	2.667E−01	6.882E−02	3.011E−02	1.642E−02	1.009E−02	6.693E−03	4.684E−03	3.412E−03
130	1.060E+01	2.490E−01	6.409E−02	2.802E−02	1.527E−02	9.383E−03	6.224E−03	4.355E−03	3.173E−03
140	1.004E+01	2.336E−01	5.998E−02	2.620E−02	1.428E−02	8.769E−03	5.816E−03	4.070E−03	2.965E−03
150	9.531E+00	2.200E−01	5.637E−02	2.461E−02	1.340E−02	8.232E−03	5.459E−03	3.820E−03	2.782E−03
160	9.078E+00	2.079E−01	5.318E−02	2.320E−02	1.263E−02	7.757E−03	5.143E−03	3.599E−03	2.621E−03
170	8.669E+00	1.971E−01	5.033E−02	2.194E−02	1.194E−02	7.334E−03	4.863E−03	3.402E−03	2.478E−03
180	8.299E+00	1.875E−01	4.778E−02	2.082E−02	1.133E−02	6.955E−03	4.611E−03	3.226E−03	2.349E−03
190	7.961E+00	1.787E−01	4.547E−02	1.980E−02	1.077E−02	6.613E−03	4.384E−03	3.067E−03	2.234E−03
200	7.652E+00	1.708E−01	4.339E−02	1.888E−02	1.027E−02	6.304E−03	4.179E−03	2.923E−03	2.129E−03
225	6.981E+00	1.538E−01	3.893E−02	1.692E−02	9.199E−03	5.645E−03	3.741E−03	2.617E−03	1.905E−03
250	6.427E+00	1.399E−01	3.531E−02	1.533E−02	8.332E−03	5.111E−03	3.387E−03	2.369E−03	1.725E−03
275	5.960E+00	1.284E−01	3.232E−02	1.402E−02	7.615E−03	4.670E−03	3.094E−03	2.164E−03	1.575E−03
300	5.561E+00	1.187E−01	2.980E−02	1.292E−02	7.012E−03	4.299E−03	2.848E−03	1.992E−03	1.450E−03
350	4.913E+00	1.032E−01	2.580E−02	1.116E−02	6.056E−03	3.712E−03	2.458E−03	1.719E−03	1.251E−03
400	4.409E+00	9.140E−02	2.276E−02	9.833E−03	5.331E−03	3.266E−03	2.163E−03	1.512E−03	1.100E−03
450	4.005E+00	8.206E−02	2.036E−02	8.788E−03	4.762E−03	2.917E−03	1.931E−03	1.350E−03	9.820E−04
500	3.674E+00	7.450E−02	1.843E−02	7.946E−03	4.303E−03	2.635E−03	1.744E−03	1.219E−03	8.870E−04
600	3.160E+00	6.299E−02	1.550E−02	6.672E−03	3.610E−03	2.209E−03	1.462E−03	1.022E−03	7.430E−04
700	2.780E+00	5.464E−02	1.339E−02	5.753E−03	3.110E−03	1.903E−03	1.259E−03	8.800E−04	6.400E−04
800	2.486E+00	4.828E−02	1.179E−02	5.058E−03	2.733E−03	1.672E−03	1.106E−03	7.720E−04	5.620E−04
900	2.252E+00	4.329E−02	1.054E−02	4.515E−03	2.438E−03	1.491E−03	9.860E−04	6.890E−04	5.010E−04
1000	2.060E+00	3.925E−02	9.527E−03	4.078E−03	2.201E−03	1.346E−03	8.900E−04	6.210E−04	4.520E−04
2000	1.141E+00	2.058E−02	4.904E−03	2.085E−03	1.122E−03	6.850E−04	4.520E−04	3.160E−04	2.290E−04
3000	8.045E−01	1.409E−02	3.325E−03	1.408E−03	7.560E−04	4.610E−04	3.040E−04	2.120E−04	1.540E−04

**Table 2:** Excitation cross sections for dipole- and spin-allowed excitations from the ground state of K. Excitation energies *E* in eV, dipole *f* values from uncorrelated wave functions (*f*_sc_), accurate *f* values (*f*_accu_) by Migdalek and Kim [24], and BE*f*-scaled excitation cross sections *σ*_BE_*_f_* in Å^2^ as functions of incident electron energy *T* in eV. The initial state is 4*s* ^2^S. The experimental ionization energy *B* = 4.340665 eV has been used in the scaling. The constants *a*, *b*, and *c* of Eq. (6) are included

Final state	4*p*	5*p*	6*p*	7*p*	8*p*	9*p*	10*p*	11*p*	12*p*
*E*	1.615E+00	3.064E+00	3.596E+00	3.853E+00	3.996E+00	4.084E+00	4.142E+00	4.183E+00	4.212E+00
*f*_sc_	1.047E+00	7.620E−03	7.240E−04	1.480E−04	4.500E−05	1.800E−05	8.000E−06	4.000E−06	2.000E−06
*f*_accu_	1.002E+00	8.220E−03	8.200E−04	1.750E−04	5.500E−05	2.200E−05	1.100E−05	6.000E−06	3.000E−06
Const. *a*	8.820E+00	3.383E−02	2.740E−03	5.240E−04	1.540E−04	5.900E−05	2.700E−05	1.400E−05	8.000E−06
Const. *b*	2.437E+01	6.465E−01	1.602E−01	6.641E−02	3.461E−02	2.055E−02	1.329E−02	9.119E−03	6.547E−03
Const. *c*	−2.041E−01	−3.857E−02	−6.663E−03	−2.013E−03	−8.140E−04	−3.950E−04	−2.170E−04	−1.300E−04	−8.400E−05
*T*	*σ*_BE_*_f_*	*σ*_BE_*_f_*	*σ*_BE_*_f_*	*σ*_BE_*_f_*	*σ*_BE_*_f_*	*σ*_BE_*_f_*	*σ*_BE_*_f_*	*σ*_BE_*_f_*	*σ*_BE_*_f_*

1.614726	0								
2.0	3.029E+01								
2.5	4.308E+01								
3.0	5.062E+01								
3.5	5.552E+01	1.251E+00							
4.0	5.880E+01	1.590E+00	3.239E−01	6.216E−02					
4.5	6.099E+01	1.747E+00	4.153E−01	1.643E−01	8.185E−02	4.718E−02	2.989E−02	2.022E−02	1.439E−02
5.0	6.243E+01	1.826E+00	4.552E−01	1.895E−01	9.918E−02	5.972E−02	3.931E−02	2.747E−02	2.009E−02
5.5	6.332E+01	1.863E+00	4.740E−01	2.011E−01	1.068E−01	6.506E−02	4.320E−02	3.040E−02	2.236E−02
6	6.381E+01	1.874E+00	4.818E−01	2.061E−01	1.102E−01	6.746E−02	4.495E−02	3.172E−02	2.338E−02
7	6.397E+01	1.855E+00	4.807E−01	2.072E−01	1.113E−01	6.840E−02	4.571E−02	3.232E−02	2.385E−02
8	6.345E+01	1.809E+00	4.694E−01	2.027E−01	1.091E−01	6.713E−02	4.490E−02	3.177E−02	2.346E−02
9	6.252E+01	1.751E+00	4.540E−01	1.962E−01	1.057E−01	6.503E−02	4.350E−02	3.079E−02	2.274E−02
10	6.137E+01	1.689E+00	4.373E−01	1.889E−01	1.018E−01	6.263E−02	4.191E−02	2.966E−02	2.190E−02
15	5.477E+01	1.403E+00	3.596E−01	1.550E−01	8.339E−02	5.130E−02	3.431E−02	2.428E−02	1.793E−02
20	4.879E+01	1.187E+00	3.018E−01	1.297E−01	6.974E−02	4.288E−02	2.867E−02	2.029E−02	1.498E−02
25	4.387E+01	1.027E+00	2.593E−01	1.112E−01	5.975E−02	3.673E−02	2.455E−02	1.737E−02	1.282E−02
30	3.984E+01	9.036E−01	2.270E−01	9.726E−02	5.221E−02	3.208E−02	2.145E−02	1.517E−02	1.120E−02
35	3.651E+01	8.069E−01	2.019E−01	8.637E−02	4.634E−02	2.847E−02	1.903E−02	1.346E−02	9.935E−03
40	3.373E+01	7.291E−01	1.817E−01	7.766E−02	4.165E−02	2.558E−02	1.710E−02	1.209E−02	8.926E−03
45	3.136E+01	6.651E−01	1.652E−01	7.054E−02	3.782E−02	2.323E−02	1.552E−02	1.098E−02	8.102E−03
50	2.932E+01	6.115E−01	1.514E−01	6.461E−02	3.463E−02	2.126E−02	1.421E−02	1.005E−02	7.416E−03
60	2.600E+01	5.270E−01	1.298E−01	5.532E−02	2.963E−02	1.819E−02	1.215E−02	8.593E−03	6.343E−03
70	2.340E+01	4.633E−01	1.137E−01	4.836E−02	2.590E−02	1.589E−02	1.062E−02	7.506E−03	5.540E−03
80	2.131E+01	4.136E−01	1.011E−01	4.296E−02	2.300E−02	1.411E−02	9.424E−03	6.663E−03	4.918E−03
90	1.959E+01	3.736E−01	9.101E−02	3.865E−02	2.068E−02	1.269E−02	8.473E−03	5.990E−03	4.421E−03
100	1.814E+01	3.409E−01	8.278E−02	3.513E−02	1.879E−02	1.153E−02	7.697E−03	5.441E−03	4.016E−03
110	1.691E+01	3.135E−01	7.593E−02	3.219E−02	1.721E−02	1.056E−02	7.050E−03	4.984E−03	3.678E−03
120	1.585E+01	2.903E−01	7.013E−02	2.971E−02	1.588E−02	9.741E−03	6.504E−03	4.598E−03	3.393E−03
130	1.492E+01	2.703E−01	6.515E−02	2.759E−02	1.475E−02	9.041E−03	6.037E−03	4.267E−03	3.149E−03
140	1.411E+01	2.529E−01	6.085E−02	2.575E−02	1.376E−02	8.436E−03	5.632E−03	3.981E−03	2.938E−03
150	1.338E+01	2.377E−01	5.708E−02	2.414E−02	1.290E−02	7.906E−03	5.278E−03	3.731E−03	2.753E−03
160	1.273E+01	2.243E−01	5.375E−02	2.272E−02	1.214E−02	7.439E−03	4.966E−03	3.510E−03	2.590E−03
170	1.215E+01	2.123E−01	5.079E−02	2.146E−02	1.146E−02	7.024E−03	4.689E−03	3.314E−03	2.446E−03
180	1.162E+01	2.016E−01	4.814E−02	2.033E−02	1.086E−02	6.654E−03	4.442E−03	3.139E−03	2.316E−03
190	1.114E+01	1.919E−01	4.576E−02	1.932E−02	1.031E−02	6.320E−03	4.219E−03	2.982E−03	2.200E−03
200	1.070E+01	1.831E−01	4.361E−02	1.840E−02	9.821E−03	6.018E−03	4.017E−03	2.839E−03	2.095E−03
225	9.750E+00	1.644E−01	3.902E−02	1.645E−02	8.775E−03	5.377E−03	3.589E−03	2.536E−03	1.871E−03
250	8.965E+00	1.492E−01	3.531E−02	1.487E−02	7.932E−03	4.859E−03	3.243E−03	2.292E−03	1.691E−03
275	8.305E+00	1.366E−01	3.225E−02	1.357E−02	7.236E−03	4.432E−03	2.958E−03	2.090E−03	1.542E−03
300	7.742E+00	1.260E−01	2.968E−02	1.248E−02	6.653E−03	4.075E−03	2.719E−03	1.921E−03	1.418E−03
350	6.831E+00	1.092E−01	2.561E−02	1.076E−02	5.730E−03	3.509E−03	2.341E−03	1.654E−03	1.220E−03
400	6.123E+00	9.641E−02	2.252E−02	9.450E−03	5.033E−03	3.081E−03	2.056E−03	1.452E−03	1.071E−03
450	5.557E+00	8.635E−02	2.011E−02	8.429E−03	4.487E−03	2.747E−03	1.832E−03	1.294E−03	9.550E−04
500	5.093E+00	7.823E−02	1.817E−02	7.608E−03	4.049E−03	2.478E−03	1.653E−03	1.168E−03	8.610E−04
600	4.376E+00	6.591E−02	1.523E−02	6.368E−03	3.387E−03	2.072E−03	1.382E−03	9.760E−04	7.200E−04
700	3.845E+00	5.700E−02	1.312E−02	5.478E−03	2.912E−03	1.781E−03	1.188E−03	8.390E−04	6.190E−04
800	3.436E+00	5.025E−02	1.152E−02	4.807E−03	2.554E−03	1.562E−03	1.042E−03	7.360E−04	5.430E−04
900	3.110E+00	4.495E−02	1.028E−02	4.283E−03	2.275E−03	1.391E−03	9.270E−04	6.550E−04	4.830E−04
1000	2.844E+00	4.069E−02	9.274E−03	3.862E−03	2.051E−03	1.254E−03	8.360E−04	5.900E−04	4.350E−04
2000	1.571E+00	2.109E−02	4.722E−03	1.955E−03	1.036E−03	6.330E−04	4.220E−04	2.980E−04	2.190E−04
3000	1.106E+00	1.436E−02	3.182E−03	1.313E−03	6.950E−04	4.240E−04	2.820E−04	1.990E−04	1.470E−04
